# Treatment of severe hospital-acquired and ventilator-associated pneumonia: a systematic review of inclusion and judgment criteria used in randomized controlled trials

**DOI:** 10.1186/s13054-017-1755-5

**Published:** 2017-06-27

**Authors:** Emmanuel Weiss, Wafa Essaied, Christophe Adrie, Jean-Ralph Zahar, Jean-François Timsit

**Affiliations:** 1 Department of Anesthesiology and Critical Care, HUPNVS, Hôpital Beaujon, APHP, 92110 Clichy, France; 2 Paris Diderot University, Paris, France; 3 Inserm UMR_S 1149 Center for Research On Inflammation Inserm/University Paris Diderot, Sorbonne Paris Cité, F-75018 Paris, France; 4 UMR 1137 - IAME Team 5 – DeSCID: Decision SCiences in Infectious Diseases, control and care Inserm/University Paris Diderot, Sorbonne Paris Cité, F-75018 Paris, France; 5 Physiology Department, APHP, Hôpital Cochin, Paris, France; 6 Microbiology and infection control unit, APHP, Hôpital Avicenne, Paris, France; 7 Medical and Infectious Intensive Care Unit, AP-HP, Hôpital Bichat Claude Bernard, Paris Diderot University, Paris, F-75018 France

**Keywords:** Systematic review, Hospital-acquired pneumonia treatment, Ventilator-acquired pneumonia treatment, Randomized controlled trials, Endpoints, Inclusion criteria

## Abstract

**Background:**

Hospital-acquired and ventilator-associated pneumonia (HAP/VAP) are often selected for randomized clinical trials (RCTs) aiming at new drug approval. Guidelines for the design of such RCTs have been repeatedly updated by regulatory agencies. We hypothesized that large variability in the enrolled populations, the endpoints assessed and the HAP/VAP definition criteria may impact the results of these studies, and addressed this through a systematic review of HAP/VAP RCTs.

**Methods:**

A search (Pubmed-Embase-ICAAC-ECCMID) of all RCTs published between 1994 and 2016 comparing antimicrobial treatment for HAP/VAP in the intensive care unit was conducted. The populations enrolled, inclusion/exclusion criteria, statistical design and endpoints assessed were recorded. All unpublished RCTs recorded on the ClinicalTrials.gov registry were also screened.

**Results:**

From the 93 abstracts reviewed, 39 potentially relevant studies were inspected, leading to 27 studies being included. As expected, illness severity or the proportion with VAP (27–100%) differed greatly among the enrolled populations. The HAP/VAP definition used various clinical and biological criteria, and only 55% of studies required a microbiological sample. The mandatory duration of prior hospital stay was variable; the mechanical ventilation duration was an inclusion criterion in only 41% of VAP studies. Nine studies had non-inferiority design, but nine studies (33%) did not have a pre-specified statistical hypothesis. Clinical cure was the primary endpoint in 24 studies, but was recorded in several populations or as the co-primary endpoint in 13 studies. The definition of clinical cure and the timing of its assessment greatly differed. This variability slightly improved over time but remained significant in the 13 registered but currently unpublished RCTs that we screened.

**Conclusion:**

Our study provides a description of populations and endpoints of RCTs evaluating antimicrobials for treatment of HAP/VAP in the ICU. There was significant heterogeneity in enrollment criteria, endpoints and statistical design, which may influence the ability of studies to demonstrate differences between studied drugs.

**Electronic supplementary material:**

The online version of this article (doi:10.1186/s13054-017-1755-5) contains supplementary material, which is available to authorized users.

## Background

New antimicrobials are required to face the dramatically increasing prevalence of multidrug-resistant pathogens. This unmet need has become a worldwide source of concern, and government action plans aiming at increasing the antimicrobials pipeline are in development [1]. The evaluation of these new treatments will require properly designed studies with appropriate inclusion criteria and endpoints. However, in hospital-acquired pneumonia (HAP) and ventilator-associated pneumonia (VAP), the way to conduct such studies remains unclear. During the past ten years, the regulatory agencies (European Medicines Agency (EMA) and Food and Drug Administration (FDA)) have repeatedly updated their guidelines for the design of randomized controlled trials (RCTs), but their recommendations remain conflicting, especially regarding the design, endpoints, or inclusion criteria that should be used [2, 3].

This is an important issue because HAP and VAP are ideal syndromes for clinical trials in the context of new drug approval: they are a major cause of infection, often involving multidrug-resistant pathogens (25% of ICU infections), they account for up to 50% of antibiotic prescriptions [4], identification of the causative pathogen using microbiological samples is easy, and they are particularly sensitive to antimicrobial treatment effect [5, 6].

Our hypothesis is that to date, HAP/VAP RCTs greatly differ in the population enrolled, the criteria used for definition of HAP/VAP, and the endpoints assessed. Such differences among studies may be of importance as they may impact the results of the studies. For example, some studies showed that the reported incidence of VAP greatly depends on the diagnostic methodology used. This systematic review describes the enrolled populations, design, and conduct of RCTs addressing the efficacy of antimicrobials for HAP/VAP treatment in intensive care unit (ICU) patients from 1994 to 2016.

## Methods

The methodology of the Preferred Reporting Items for Systematic Reviews and Meta Analyses (PRISMA) guidelines was used for the conduct of the systematic review [7].

### Search Strategy

We searched PubMed, Embase, ICAAC and ECCMID for publications from 1994 to 2016. The search string used was:

1-(Pneumonia and (hospitalized or acquired or ventilatory-associated or nosocomial or health-associated)) and (antibiotic treatment or (antibiotic therapy) or antibiotic or (antibacterial agents) or (anti-infective agents) or antimicrobial)

It was complemented by:

2-Pneumonia and (hospitalized or acquired or ventilatory-associated or nosocomial or health-associated)) and (doripenem or meropenem or impenem or linezolid or vancomycin or piperacillin or (piperacillin and tazobactam) or ceftazidim or ceftriaxone or cefotaxime or ceftobiprole or ciprofloxacin or levofloxacin or moxifloxacin or piperacillin or teicoplanin or cefepime or ticarcillin or (ticarcillin and clavulanic acid) or amikacin or tobramycin or gentamicin) and (randomized controlled trial).

Of note, some studies were identified through sources other than a database search, including contact with researchers.

### Study selection

**Fig. 1:**
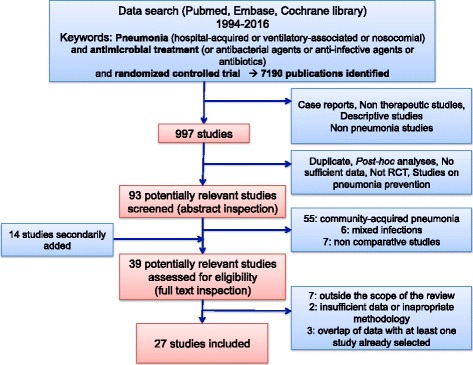
Study selection flow diagram

All RCTs evaluating the efficacy of antimicrobials in HAP/VAP in adult patients in the ICU from 1994 to 2016 were considered as eligible for this systematic review. The study selection process is detailed in a flow diagram (Fig. 1). The search period was limited to twenty years because of the evolution of publication requirements and treatment concepts. All articles were independently screened by two authors (J-RZ and CA). Full texts (and clinical trial registry data (ClinicalTrials.gov website)) of all potentially relevant studies were analyzed to assess eligibility. Disagreements on the inclusion/exclusion of studies were resolved in consultation with other co-authors (EW and J-FT). We included studies comparing one antibiotic agent to another, monotherapy to combination therapy, inhaled to systemic route or extended/continuous infusion to intermittent administration. Finally, in January 2017 we also searched the ClinicalTrials.gov website for characteristics of currently unpublished RCTs comparing HAP/VAP antimicrobial treatment strategies.

### Recorded data

The following data were collected for each study included: inclusion and exclusion criteria, design, primary endpoint and population in which it was analyzed, definition of clinical cure, time point used for the test of cure (TOC) visit, and the statistical hypothesis. Data extraction from the studies was confirmed by an independent reviewer. At the end, a global quality assessment of each RCT was performed using an 8-item scale allowing calculation of a global quality score adapted to the focus of this systematic review.

## Results

### Populations enrolled

The main characteristics of the 27 studies included in the systematic review (see Additional file 1 for references) and of their enrolled populations are described in Table 1.

**Table 1:** Selected characteristics of studies included in the systematic review

First author	Year of publication	Tested molecule	Comparator	Statistical design	Blinded	Causative pathogen	Patients with VAP (ITT or mITT, %)	Number of ITT patients	Patient with positive respiratory samples (% of ITT/primary endpoint population)	Mean age ± SD^a^	Mean Apache II score ± SD^a^	Duration of study therapy, (days)	Cure rate (%)	Mortality rate (%)
Jaccard	1998	Piperacillin/tazobactam	Imipenem/cilastatin	NM	Yes	All pathogens	NM	154	81/81	59.7 ± 16.9	14.9 ± 6.8	7–14	77	8.4^l^
Brun-Buisson	1998	Piperacillin/tazobactam + amikacin	Ceftazidim + amikacin	Equivalence	No	All pathogens	100	204	62/100	55.5 ± 16	37.4 ± 1.4^f^	15–21	42	20^i^
Fagon	2000	Quinupristin/dalfopristin	Vancomycin	Equivalence	No	Only Gram-positive pathogens	70	304	73/100	57 ± 18	15 ± 6.9	5–14	44	23^k^
Torres	2000	Ciprofloxacin	Imipenem/cilastatin	NM	No	All pathogens	NM	149	68/100	61 ± 16	13.8 ± 8	NM	68	16^k^
Alvarez-Lerma	2001	Piperacillin/tazobactam + amikacin	Ceftazidim + amikacin	Equivalence	No	All pathogens	85	124	56/64	59 ± 19	16.7 ± 6.5	NM	48	35^k^
Nicolau	2001	Continuous ceftazidime	Intermittent ceftazidime	NM	No	All pathogens	91	35	68/80	51 ± 18	14.5 ± 5.5	NM	37	NM
Rubinstein	2001	Linezolid	Vancomycin	Equivalence	Yes	All pathogens	63	396	42//NM	62 ± 18	15.5 ± 6.8	7	52	21^k^
Wunderlink	2003	Linezolid	Vancomycin	Equivalence	Yes	All pathogens	35	623	71//100	62.5 ± 19.1	14.1 ± 6	7–21	53	20^j^
Zanetti	2003	Cefepim	Imipenem/cilastatin	Non inferiority	Yes	All pathogens	NM	209	NM/71	54 ± 18	15 ± 6.5	NM	39	22^i^
Shorr	2005	Levofloxacin	Imipenem/cilastatin	Non inferiority	No	All pathogens	100	222	>50/>50	53 ± 21	15 ± 14	NM	61 ^g^	NM
Joshi	2006	Piperacillin/tazobactam + tobramycin	Imipenem/cilastatin + tobramycin	Equivalence	Yes	All pathogens	69	437	92//100	52.3 ± 20	13.5	>5	53	NM
Schmidt	2006	Piperacillin/tazobactam	Imipenem/cilastatin	NM	Yes	All pathogens	NM	221	100/100	67 ± 13.7	13.4 ± 4.2	5–21	68 ^g^	13^k^
Betrosian	2008	Ampicillin-sulbactam	Colistin	NM	No	*Acinetobacter baumanii*	100	28	100/100	70 ± 7	14 ± 3.5	8–10	68	28^h^
Chastre	2008	Doripenem	Imipenem/cilastatin	Non inferiority	No	All pathogens	100	531	78/82	50,5 ± 19	RS 59 < 15	7–14	58	10^h^
Giamarallos-Bourboulls	2008	ATB + clarithromycin	ATB + placebo	NM	yes	All pathogens	100	200	100/100	58.4 ± 18.4	17 ± 6	NM	75	25^i^
Heyland	2008	Meropenem + ciprofloxacin	Meropenem	Superiority	no	All pathogens	100	740	82/82	59 ± 17.8	20 ± 6.3	NM	NM	19^h^
Freire	2010	Tigecyclin	Imipenem/cilastatin	Non inferiority	yes	All pathogens	26.8	945	65/62	57.4 ± 18.6	RS 396 > 15	7–14	52	13^k^
Jung	2010	Vancomycin + rifampicin	Vancomycin	Superiority	no	Methicillin-resistant *Staphylococcus aureu*s	73	83	100/100	69 (28-98)^b^	24 (15–38)^b^	14	42	30^h^
Rattanaum-pawan	2010	ATB + nebulized colistimetate	ATB + placebo	Superiority	no	Only Gram-negative pathogens	100	102	100/100	68 ± 16.5	18.7 ± 5	NS	52	42^h^
Lu	2011	Nebulized ceftazidime + amikacin	IV ceftazidim + amikacin	NM	No	*Pseudomonas aeruginosa*	100	40	100/100	59 ± 16	33 ± 13^f^	8	72	18^h^
Rubinstein	2011	Telavancin	Vancomycin	Non inferiority	Yes	Only Gram-positive pathogens	28	1503	60/73	62 ± 18	15.5 ± 6.2	7–21	59	19^h^
Aydemir	2012	Colistin	Colistin + rifampicin	NM	No	*Acinetobacter baumanii*	100	43	100/100	61 ± 20	19.1 ± 6	NS	46	67^k^
Kollef	2012	Doripenem	Imipenem	Non inferiority	Yes	All pathogens	100	227	74/100	54.7 ± 17.6	RS^e^	7–10	52	18^h^
Wunderlink	2012	Linezolid	Vancomycin	Non inferiority	Yes	*Staphylococcus aureus*	63	448	100/100	61 ± 18	17 ± 6	7–14	50	16^j^
Ramirez	2013	Tigecyclin Low dose/Tigecyclin High dose	Imipenem/cilastatin	Non inferiority	Yes	All pathogens	39	105	63/71	62 ± 15	13.8	NS	59	16^k^
Awad	2014	Ceftobiprol medocaril	Ceftazidim + vancomycin	Non inferiority	Yes	All pathogens	27	781	69/NM	RS^c^	RS^d^	7–14	51	17^i^
Kollef	2016	Imipenem or meropenem + Nebulized amikacin and fosfomycin	Imipenem or meropenem + placebo	NM	Yes	Only Gram-negative pathogens	100	143	100/100	62 ± 11	18.4 ± 5.9	10	23	20

*APACHE* Acute Physiology and Chronic Health Evaluation, *ITT* intention-to-treat, *mITT* microbiological ITT, *NM* not mentioned, *RS* reported by stratum only, *SAPS* Simplified Acute Physiology Score, *SD* standard deviation, *VAP* ventilator-associated pneumonia

^a^As provided in reports

^b^Median (range)

^c^362 patients were >65 years old

^d^205 patients had an APACHE II score >15

^e^128 patients had an APACHE II score >15

^f^Mean ± SD SAPS

^g^Clinical success rate (include clinical cure and clinical improvement)

^h^Day-28 mortality

^i^Day-30 mortality

^j^Day-60 mortality

^k^Mortality at the end of the study

^l^Infection-related mortality

The number of patients enrolled in the intention-to-treat (ITT) population was highly variable and ranged from 35 to 945. Patients were predominantly male and the mean age across trials was 50.5–70.0 years. In most studies (92%), severity of illness was reported using the APACHE II score and its mean value varied from 13.8 to 24.0 across trials. The rate of comorbidity was rarely reported.

Sixteen studies co-enrolled patients with HAP and VAP and 11 studies exclusively enrolled patients with VAP. Among the former, the proportion of patients with VAP greatly varied from 27 to 91%. In some studies, randomization was stratified by patients’ severity of illness (three trials) and on the rate of VAP (two trials). Whereas 18 studies (67%) included all HAP/VAP episodes regardless of the causative pathogen, some only enrolled patients with HAP/VAP related to specific pathogens. The duration of hospital stay before the onset of pneumonia was required to be longer than 48 hours and 72 hours in 18 studies (67%) and 7 studies (26%), respectively (not mentioned in the remaining study). However, the duration of the mechanical ventilation was an inclusion criterion in only 11 VAP studies (41%).

**Fig. 2:**
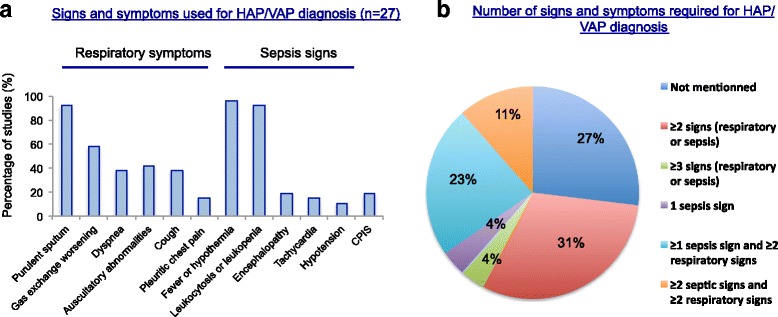
Variability of signs and symptoms used for definition of hospital-acquired pneumonia/ventilator-associated pneumonia (*HAP/VAP*) (n = 27 studies). **a** Frequency of signs and symptoms used as HAP/VAP diagnostic criteria. **b** Number of signs and symptoms required for HAP/VAP diagnosis. *CPIS* Clinical Pulmonary Infection Score

The diagnosis of pneumonia was consistently based on clinical and radiological (new lung infiltrate on chest radiography) findings but microbiological confirmation was required in only 15 studies (55%). Among the remaining studies, the proportion of patients analyzed who had positive respiratory samples varied from 62 to 82%. Clinical signs leading to suspicion of HAP/VAP were highly variable across the 27 studies. These clinical findings and the percentage of studies using them as inclusion criteria are shown in Fig. 2a. In 73% of studies, a predefined number of respiratory symptoms and signs of sepsis were required for pneumonia diagnosis and subsequent inclusion (Fig. 2b).

Various exclusion criteria were also reviewed. In five studies (20%), patients with severe disease, as reflected by high Acute Physiology and Chronic Health Evaluation (APACHE) score (four studies) or Simplified Acute Physiology Score (SAPS) (one study), concomitant septic shock (two studies) or acute respiratory distress syndrome (one study) were excluded. Neutropenia and immunosuppression (various definitions) were considered as exclusion criteria in 13 studies (48%) and 7 studies (26%), respectively. Finally, patients with chronic respiratory diseases were excluded in nine studies (33%).

Only a few studies tried to balance heterogeneity by stratifying the randomization according to the severity of the acute disease (APACHE in two studies and partial arterial oxygen pressure/fraction of inspired oxygen (PaO2/FiO2) ratio in one study) or the characteristics of the pneumonia (i.e., HAP or VAP, in three studies).

### Clinical trial design

There were 21 multicenter studies (78%), 14 double-blinded studies (52%) and 13 open-labeled studies (48%). The duration of study therapy varied from one study to another (from 5 to 21 days) and was left at the discretion of the investigators in 37% of studies.

Nine trials (33%) used a non-inferiority design with non-inferiority margins (i.e., absolute percentage difference in the primary outcome acceptable for non-inferiority to be established) mentioned in eight of them (20%, 15% and 10% in three studies, four studies, and one study, respectively), but the scientific evidence leading to calculation of the non-inferiority margin was missing in two of them. Six studies (22%) and three studies (11%) were equivalence and superiority trials, respectively. Of note, a pre-specified statistical hypothesis was lacking in nine studies (33%) and sample size calculation was not mentioned in nine studies (33%).

### Primary endpoint

Our work revealed marked heterogeneity among the primary endpoints reported in HAP/VAP trials. A single primary endpoint in one population was reported in 13 studies (48%): a clinical cure in 12 trials (44%), CPIS decrease in 1 trial (4%), and 28-day mortality in 1 (4%).

**Fig. 3:**
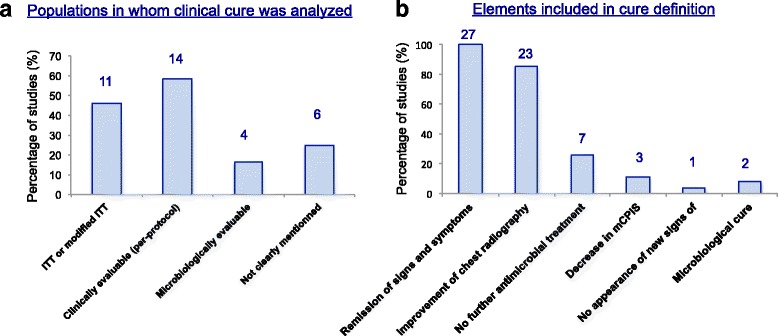
Clinical cure analysis. **a** Populations in whom clinical cure was analyzed (n = 24 studies. *ITT* intention-to-treat. **b** Items included in the definition of clinical cure. *mCPIS* modified Clinical Pulmonary Infection Score

In seven studies, the clinical cure was analyzed in several populations (two populations in six studies, and three populations in one study) without specifying which one was the primary endpoint. In four studies, the clinical cure and microbiological cure were both reported as the primary endpoint and safety was also added in two studies. Altogether, clinical cure was the primary endpoint (or one of the “primary” endpoints) in 24 studies (89%) and mortality in only 2 studies. The various populations used to analyze clinical cure are displayed in Fig. 3a.

For a global assessment of study quality, we calculated an 8-point quality score based on 8 systematically evaluated items, reflecting enrollment criteria and methodology (Table 2). Altogether, this score ranged from 2 to 7, thereby confirming the heterogeneity between studies.

### Definition of clinical cure and timing of the assessment

Interestingly, elements included in the definition of clinical cure greatly differed among studies (Fig. 3b). Remission of signs and symptoms of pneumonia were included in the definition of clinical cure in all studies, but was only partial in 11 (41%) studies. Chest radiography findings were not systematically taken into account (22 studies, 82%) and they qualified for clinical cure whether the improvement was complete, partial or only a lack of progression in 18 studies (67%). Finally, no need for additional antibiotics during follow up and decrease in the Clinical Pulmonary Infection Score (CPIS) [8] were used in 26% and 11% of studies, respectively. The timing of the TOC visit was different across trials. The time point was between day 7 and day 21 after the end of therapy in 56% percent of the studies; in the remaining trials, it ranged from the end of treatment (4 studies, 15%) till day 30 after treatment (3 studies, 12%). These differences in the definition of clinical cure and in the population and time points used to assess it led to wide variability in reported clinical cure rates that ranged from 23 to 77% (Table 1).

### Mortality

All-cause mortality was reported in 22 studies (81%) (primary endpoint in one study) and HAP/VAP-related mortality was reported in 2 studies (Table 1). The time points used to assess mortality were highly variable: day 28 in nine studies, at the end of the study in eight studies, and day 30 and day 60 in four studies and two studies, respectively. At day 28, the mean mortality rate was 23%, with broad variability of the reported all-cause mortality rates from one study to another (range 10–67%; Table 1).

### Improvement in methodological quality over time

The methodological quality of studies improved over time. We specifically analyzed the six studies published after the EMA workshop on antimicrobials held in 2011, i.e., between 2012 and 2016. Among the six studies, four had a non-inferiority design (with anticipated statistical approach and non-inferiority margin), were double-blinded, and assessed clinical cure in one population as the single primary endpoint. Clinical criteria for diagnosis of HAP/VAP were more homogenous and at least two clinical signs were consistently required. Furthermore, all cases of pneumonia that were analyzed were microbiologically proven in four of the six trials. Finally, the only items that did not improve were the definition of clinical cure and the time points used to assess it. Calculation of the mean quality score confirmed this global improvement: the mean score of studies published after 2011 was higher than that of older studies (5.3 vs 3.9) (Table 2).

### Features of currently unpublished RCTs reported on ClinicalTrials.gov. comparing antimicrobial treatment strategies in HAP/VAP

Thirteen currently unpublished RCTs that tested new antimicrobial treatment strategies in patients with HAP/VAP or with severe infections due to carbapenem-resistant bacteria including HAP/VAP are registered on ClinicalTrials.gov (Table 3)*.* Interestingly, the primary endpoint remained highly variable: 28-day all-cause mortality in four studies (all comparing systemic molecules) and clinical cure (with a variable definition, and using variable timing of assessment) in nine studies (four of them comparing systemic molecules and five testing the efficacy of nebulized antimicrobials) and a favorable clinical response. Only five studies described which population was analyzed.

**Table 2:**
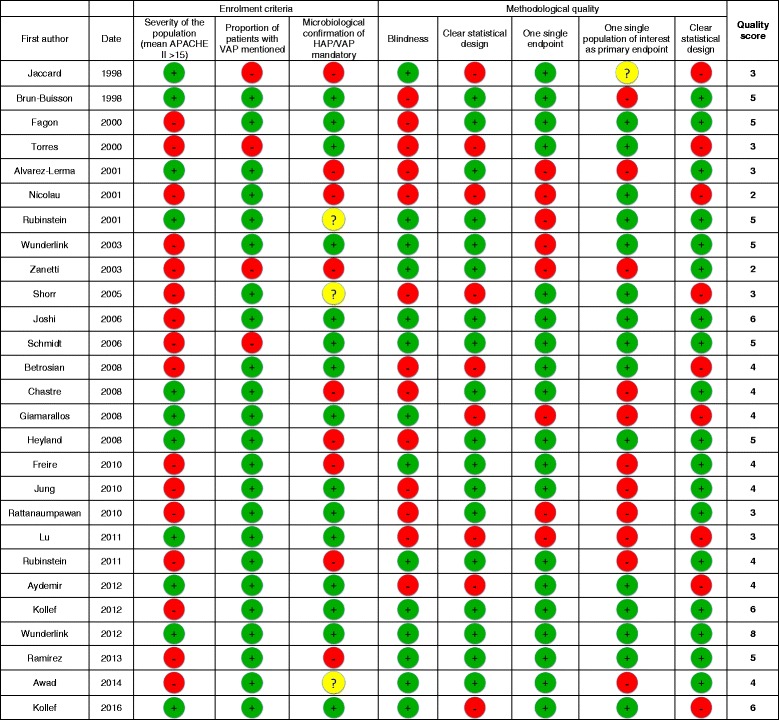
Quality assessment of the included randomized controlled trials using eight items and allowing calculation of a quality score

**Table 3:** Characteristics of currently unpublished RCTs reported on ClinicalTrials.gov. website, comparing antimicrobials in the management of HAP/VAP

ClinicalTrial.gov identifier	Investigational agent	Comparator	Name of the study	Condition	Primary outcome	Definition of clinical cure	Primary endpoint assessment timing	Analyzed populations	Current status of the study
NCT02714595	S-649266	Best available therapy	CREDIBLE	Severe infections due to carbapenem-resistant GNB including HCAP, HAP and VAP	Clinical cure	1) Resolution or improvement of baseline signs of pneumonia	Day 7 after EOT	Not reported	Recruiting
						2) Improvement of chest radiography			
						3) No additional antibacterial therapy required for current infection treatment			
NCT02070757	Ceftolozane/	Meropenem	ASPECT-NP	VAP or ventilated HAP	All-cause mortality	Not detailed	Day 28	ITT	Recruiting
	Tazobactam								
NCT01808092	Ceftazidime/	Meropenem	REPROVE	HAP/VAP	Clinical cure	1) Patient alive	Day 21 to day 25 after randomization	Co-primary:	Completed-has results
	Avibactam					2) Resolution or improvement of all signs of pneumonia		- clinically ITT	
								- clinically evaluable	
NCT02452047	Imipenem	Imipenem + cilastatin	RESTORE-IMI 1	Severe infections due to Imipenem-resistant bacteria including HAP/VAP	Favorable clinical success	Not specified	Up to day 28	Not reported	Recruiting
	+ Cilastatin/	+Colistimethate sodium							
	Relebactam								
NCT02493764	Imipenem	Piperacillin/tazobactam	RESTORE-IMI 2	HAP/VAP	All-cause mortality	Not specified	Day 28	Not reported	Recruiting
	+Cilastatin/								
	Relebactam								
NCT01970371	Plazomicin (+meropenem or tigecyclin)	Colistin	CARE	Infections due to Carbapenem-resistant *Enterobacteriaceae* including HAP and VAP	All-cause mortality	Not specified	Day 28	Not reported	Completed
		+Meropenem or Tigecyclin							
NCT02168946	Meropenem	Best available therapy	Not reported	Infections due to Carbapenem-resistant *Enterobacteriaceae* including HAP and VAP	Clinical cure	Resolution or improvement of the baseline signs and symptoms and no further antimicrobial warranted	Days 12–23	Microbiological ITT	Recruiting
	Vaborbactam								
NCT02019420	Tedizolid phosphate	Linezolid	MK-1986-002	Gram-positive HAP/VAP	All-cause mortality	Not specified	Day 28	Microbiological ITT	Recruiting
NCT02440828	Standard treatment + inhaled tobramycin	Standard treatment + Placebo	VAPORISE	VAP	Clinical response	1) Improvement of hypoxemia,	After 72 hours of treatment	Not reported	Recruiting
						2) Resolution of signs of sepsis			
						3) No worseming of chest radiography			
NCT02478710	Standard treatment + inhaled tobramycin or inhaled vancomycin	Standard treatment + Placebo	AAINTVAP	VAP	Persistance/recurrence of pneumonia	Not specified	After 8 days of treatment/day 9 to day 21	Not reported	Recruiting
NCT02728518	Standard treatment + nebulized amikacin	Standard treatment + intravenous amikacin	Not reported	GNB-related VAP	Clinical cure	Not specified	Not reported	Not reported	Completed
NCT02574130	Standard treatment + nebulized amikacin	Standard treatment + placebo	Not reported	GNB-related VAP	Clinical cure	No worsening on chest radiography + improvement of leukocytosis and/or fever and/or tracheal secretion	10 days after treatment	Not reported	Recruiting
NCT01799993	Standard treatment + amikacin inhalation solution (BAY41-6551)	Standard treatment + placebo	INHALE-1 and 2	GNB-related mechanically ventilated pneumonia	Clinical success	Not specified	28–32 days after treatment initiation	Modified ITT	Recruiting
and									
NCT00805168									

*RCT* randomized controlled trial*, HAP* hospital-acquired pneumonia, *VAP* ventilator-associated pneumonia, *ITT* intention-to-treat, *GNB* Gram-negative, *HCAP* Healthcare-associated pneumonia bacilli, *EOT* end of treatment

## Discussion

### Main findings

In this systematic review comprising 27 published RCTs addressing the efficacy of antimicrobials for treatment of HAP/VAP in critically ill patients, we found that the enrolled populations, clinical trial design, and endpoints assessed vary greatly between studies. This variability is intuitive but its quantification is of importance because, as shown by the variable mortality and clinical cure rates that were reported, it impacted the results of RCTs. This heterogeneity may therefore influence the ability of studies to demonstrate differences between investigational drugs and comparators. These results are not surprising as far as there are even differences in the guidance from regulatory agencies on how to evaluate treatments for HAP/VAP [2, 3], especially with regards to the study design, population and endpoint.

### Enrolled populations

In parallel with the inconsistent recommendations of the regulatory agencies on methodology for the diagnosis of HAP/VAP, we found great variability in the clinical criteria used for clinical diagnosis of HAP/VAP. These discrepancies were probably increased by the co-enrollment of patients with HAP and VAP in the majority of studies. Indeed, in agreement with the FDA, but not with the EMA guidelines, no distinction was made between clinical signs and symptoms used for the diagnosis of these different diseases; a minimal duration of mechanical ventilation was required as an inclusion criterion in only 41% of studies. Such a merge of populations with HAP and VAP could mask differences between groups in drug activity, and lead to false conclusions [9]. According to several authors, a combination of clinical and radiological criteria should be used to increase the pretest probability of disease before confirmatory microbiology culture for diagnosis of HAP and VAP [8, 10]. However, in our review, the number of required clinical and biological symptoms greatly varied, and scores combining clinical, biological, and microbiological data such as CPIS [8] were almost never used.

Similarly, in the context of HAP/VAP treatment trials, microbiological confirmation of pneumonia is left optional by the EMA; it was used as inclusion criterion in only 55% of studies included in our review, and among the remaining studies the proportion of microbiologically documented pneumonia varied between 60 and 80%. As a consequence, some patients without pneumonia may have been enrolled in some studies and may have led to false negative results in superiority trials and false conclusions of similarity of drugs in non-inferiority trials. Finally, the severity scores of enrolled patients were very different from one study to another and, in some studies the most severe patients were excluded. The lack of inclusion of some subgroups of patients may be questionable as far as they may specifically receive the maximum benefit of the tested antibiotic [11, 12]. Stratification of randomization of patients based on the severity of illness unfortunately was rarely used.

### Design

We identified a large proportion of studies with methodological issues. Although drug dose monitoring or use of multiple antibacterial agents may complicate double-blinding in HAP/VAP studies, it remains crucial to minimize post-randomization bias. In our review, double-blinding was performed in only half of the studies. Furthermore, a pre-specified statistical hypothesis was lacking in more than one study out of four, and the sample size calculation was not mentioned in more than one out of three. Of note, only three studies had superiority design. A statistical hypothesis of non-inferiority was three times more frequent and increased over time (four out of the five most recent studies used it). The use of this design may be the result of FDA and EMA recommendations that describe this design as “acceptable” [2, 3]. Nevertheless, non-inferiority trials are complex to conduct. They require an appropriately selected narrow non-inferiority margin and consequently a large sample size to prevent false conclusions of non-inferiority of the tested drugs. In this context, according to the FDA, mortality non-inferiority margins should be less than 10% [2]. According to the EMA, clinical cure non-inferiority margins should be less than 12.5% [3]. In our study, clinical cure non-inferiority margins were frequently mentioned but they were larger than those recommended in almost all studies, which is clinically unacceptable.

### Endpoints

The question of the best primary efficacy endpoint to use in HAP/VAP trials remains highly debatable [13]. Proof of this is provided by the conflicting guidelines provided by the regulatory agencies, with the EMA recommending the clinical outcome at the TOC visit (ranging from 14 to 21 days after the end of therapy) and the FDA recommending all-cause mortality at day 28 [2].

Our results show that the assessment of clinical response (clinical cure) by investigators was used in the vast majority of HAP/VAP RCTs. The main advantage of this criterion is its routine use by clinicians to assess patient response to an antimicrobial treatment. However, as demonstrated by the wide range of clinical cure rates showed in this review, the variable definitions of clinical cure that are used are problematic and may impact the reliability of this endpoint. These results suggest that a consensual definition of clinical cure is urgently needed. Such consensus should also homogenize the timing used to assess the primary endpoint, in order to limit its variability that may also account for variable clinical cure rates. In our review, whatever the endpoint used (i.e., clinical cure or mortality), there was wide variability in the time point used to assess it. Finally, the documentation of the magnitude of treatment effect on clinical cure should also be established. Indeed, a previous demonstration of the superiority of the comparator drug to a placebo or no therapy is necessary before conducting non-inferiority trials [14], and in HAP/VAP such benefit of effective antibacterial therapy has only been established for all-cause mortality [15] and not for clinical cure.

For this reason, mortality is the only endpoint that should be used in non-inferiority trials. However, while mortality is the endpoint that reflects the strongest outcome criteria, its choice may also offer some disadvantages. First, the mortality attributable is difficult to determine; all-cause mortality may be related to underlying comorbidity, affecting the relationship between the efficiency of the antimicrobial treatment and death. Second, low mortality rates reported in HAP/VAP trials (except for those enrolling patients with difficult-to-treat pathogens) and the large non-inferiority margins that are recommended may prevent rejection of the non-inferiority of the tested drug and biased the results.

Nevertheless, the latest FDA recommendations [2] may promote the use of mortality as a primary endpoint. Indeed, among the five currently unpublished RCTs of new systemic molecules that are reported on the ClinicalTrials.gov website, two use 28-day all-cause mortality as the primary endpoint, and one includes death in the definition of clinical cure. Clinical cure was used in seven studies, including one addressing the effect of inhaled antimicrobials as adjunctive therapy. As expected, as a clear consensus is still lacking, the variability in the definition of clinical cure and in the time point used to assess it remain significant.

## Conclusion

Altogether, this review provides a description of populations and endpoints of RCTs evaluating antimicrobials for treatment of HAP/VAP in the ICU. Our results show significant heterogeneity in the enrollment criteria, endpoints and statistical design that may influence the ability of studies to demonstrate differences between the drugs studied. Although the methodological quality of studies seems to improve over time, some pitfalls remain. In particular, as demonstrated by the variability observed in currently unpublished RCTs testing new molecules, the regulatory agencies should agree on the best primary endpoint and the timing of its assessment. In this context, composite and/or hierarchical endpoints including both mortality and clinical cure may be of particular interest, and new methodologies helping assess the risks and benefits of new antimicrobial treatment strategies such as desirability of outcome ranking (DOOR) and response adjusted for duration of antibiotic risk (RADAR) may be used [16]. Some other promising new tools such as hierarchical nested design (combining non-inferiority and nested superiority trials) or competing event analyses (considering the influence of timing of events on effect measures) have also been recently proposed to improve the design and the analysis of future trials [13]. However, some basic concepts such as clinical cure remain to be better defined.

## Additional file

Additional file 1: References for studies included in the systematic review. (DOCX 18 kb)

## Abbreviations

APACHE: Acute Physiology and Chronic Health Evaluation
CPIS: Clinical Pulmonary Infection Score
DOOR: Desirability of outcome ranking
EMA: European Medicines Agency
FDA: Food and Drug Administration
HAP: Hospital-acquired pneumonia
ICU: Intensive care unit
ITT: Intention to treat
RADAR: Response adjusted for duration of antibiotic risk
RCT: Randomized controlled trial
SAPS: Simplified Acute Physiology Score
TOC: Test of cure
VAP: Ventilator-associated pneumonia
