# Supplementary material for: Treatment of severe hospital-acquired and ventilator-associated pneumonia: a systematic review of inclusion and judgment criteria used in randomized controlled trials
Source: Crit Care. 2017 Jun 27;21:162. doi: 10.1186/s13054-017-1755-5 (PMC5488424; doi:10.1186/s13054-017-1755-5)
Supplement: Additional file 1: — References for studies included in the systematic review. (DOCX 18 kb) [file 13054_2017_1755_MOESM1_ESM.docx]

**Additional file 1**

**References of studies included in the systematic review**

1. Jaccard C, Troillet N, Harbarth S, Zanetti G, Aymon D, Schneider R, Chiolero R, Ricou B, Romand J, Huber O *et al*: **Prospective randomized comparison of imipenem-cilastatin and piperacillin-tazobactam in nosocomial pneumonia or peritonitis**. *Antimicrob Agents Chemother* 1998, **42**(11):2966-2972.

2. Brun-Buisson C, Sollet JP, Schweich H, Briere S, Petit C: **Treatment of ventilator-associated pneumonia with piperacillin-tazobactam/amikacin versus ceftazidime/amikacin: a multicenter, randomized controlled trial. VAP Study Group**. *Clin Infect Dis* 1998, **26**(2):346-354.

3. Fagon J, Patrick H, Haas DW, Torres A, Gibert C, Cheadle WG, Falcone RE, Anholm JD, Paganin F, Fabian TC *et al*: **Treatment of gram-positive nosocomial pneumonia. Prospective randomized comparison of quinupristin/dalfopristin versus vancomycin. Nosocomial Pneumonia Group**. *Am J Respir Crit Care Med* 2000, **161**(3 Pt 1):753-762.

4. Torres A, Bauer TT, Leon-Gil C, Castillo F, Alvarez-Lerma F, Martinez-Pellus A, Leal-Noval SR, Nadal P, Palomar M, Blanquer J *et al*: **Treatment of severe nosocomial pneumonia: a prospective randomised comparison of intravenous ciprofloxacin with imipenem/cilastatin**. *Thorax* 2000, **55**(12):1033-1039.

5. Alvarez-Lerma F, Insausti-Ordenana J, Jorda-Marcos R, Maravi-Poma E, Torres-Marti A, Nava J, Martinez-Pellus A, Palomar M, Barcenilla F, Spanish Collaborative Group for the Study of Severe I: **Efficacy and tolerability of piperacillin/tazobactam versus ceftazidime in association with amikacin for treating nosocomial pneumonia in intensive care patients: a prospective randomized multicenter trial**. *Intensive Care Med* 2001, **27**(3):493-502.

6. Nicolau DP, McNabb J, Lacy MK, Quintiliani R, Nightingale CH: **Continuous versus intermittent administration of ceftazidime in intensive care unit patients with nosocomial pneumonia**. *Int J Antimicrob Agents* 2001, **17**(6):497-504.

7. Rubinstein E, Cammarata S, Oliphant T, Wunderink R, Linezolid Nosocomial Pneumonia Study G: **Linezolid (PNU-100766) versus vancomycin in the treatment of hospitalized patients with nosocomial pneumonia: a randomized, double-blind, multicenter study**. *Clin Infect Dis* 2001, **32**(3):402-412.

8. Wunderink RG, Cammarata SK, Oliphant TH, Kollef MH, Linezolid Nosocomial Pneumonia Study G: **Continuation of a randomized, double-blind, multicenter study of linezolid versus vancomycin in the treatment of patients with nosocomial pneumonia**. *Clin Ther* 2003, **25**(3):980-992.

9. Zanetti G, Bally F, Greub G, Garbino J, Kinge T, Lew D, Romand JA, Bille J, Aymon D, Stratchounski L *et al*: **Cefepime versus imipenem-cilastatin for treatment of nosocomial pneumonia in intensive care unit patients: a multicenter, evaluator-blind, prospective, randomized study**. *Antimicrob Agents Chemother* 2003, **47**(11):3442-3447.

10. Shorr AF, Zadeikis N, Jackson WL, Ramage AS, Wu SC, Tennenberg AM, Kollef MH: **Levofloxacin for treatment of ventilator-associated pneumonia: a subgroup analysis from a randomized trial**. *Clin Infect Dis* 2005, **40 Suppl 2**:S123-129.

11. Joshi M, Metzler M, McCarthy M, Olvey S, Kassira W, Cooper A: **Comparison of piperacillin/tazobactam and imipenem/cilastatin, both in combination with tobramycin, administered every 6 h for treatment of nosocomial pneumonia**. *Respir Med* 2006, **100**(9):1554-1565.

12. Schmitt DV, Leitner E, Welte T, Lode H: **Piperacillin/tazobactam vs imipenem/cilastatin in the treatment of nosocomial pneumonia--a double blind prospective multicentre study**. *Infection* 2006, **34**(3):127-134.

13. Betrosian AP, Frantzeskaki F, Xanthaki A, Douzinas EE: **Efficacy and safety of high-dose ampicillin/sulbactam vs. colistin as monotherapy for the treatment of multidrug resistant Acinetobacter baumannii ventilator-associated pneumonia**. *J Infect* 2008, **56**(6):432-436.

14. Chastre J, Wunderink R, Prokocimer P, Lee M, Kaniga K, Friedland I: **Efficacy and safety of intravenous infusion of doripenem versus imipenem in ventilator-associated pneumonia: a multicenter, randomized study**. *Crit Care Med* 2008, **36**(4):1089-1096.

15. Giamarellos-Bourboulis EJ, Pechere JC, Routsi C, Plachouras D, Kollias S, Raftogiannis M, Zervakis D, Baziaka F, Koronaios A, Antonopoulou A *et al*: **Effect of clarithromycin in patients with sepsis and ventilator-associated pneumonia**. *Clin Infect Dis* 2008, **46**(8):1157-1164.

16. Heyland DK, Dodek P, Muscedere J, Day A, Cook D, Canadian Critical Care Trials G: **Randomized trial of combination versus monotherapy for the empiric treatment of suspected ventilator-associated pneumonia**. *Crit Care Med* 2008, **36**(3):737-744.

17. Freire AT, Melnyk V, Kim MJ, Datsenko O, Dzyublik O, Glumcher F, Chuang YC, Maroko RT, Dukart G, Cooper CA *et al*: **Comparison of tigecycline with imipenem/cilastatin for the treatment of hospital-acquired pneumonia**. *Diagn Microbiol Infect Dis* 2010, **68**(2):140-151.

18. Jung YJ, Koh Y, Hong SB, Chung JW, Ho Choi S, Kim NJ, Kim MN, Choi IS, Han SY, Kim WD *et al*: **Effect of vancomycin plus rifampicin in the treatment of nosocomial methicillin-resistant Staphylococcus aureus pneumonia**. *Crit Care Med* 2010, **38**(1):175-180.

19. Rattanaumpawan P, Lorsutthitham J, Ungprasert P, Angkasekwinai N, Thamlikitkul V: **Randomized controlled trial of nebulized colistimethate sodium as adjunctive therapy of ventilator-associated pneumonia caused by Gram-negative bacteria**. *J Antimicrob Chemother* 2010, **65**(12):2645-2649.

20. Lu Q, Yang J, Liu Z, Gutierrez C, Aymard G, Rouby JJ, Nebulized Antibiotics Study G: **Nebulized ceftazidime and amikacin in ventilator-associated pneumonia caused by Pseudomonas aeruginosa**. *Am J Respir Crit Care Med* 2011, **184**(1):106-115.

21. Rubinstein E, Lalani T, Corey GR, Kanafani ZA, Nannini EC, Rocha MG, Rahav G, Niederman MS, Kollef MH, Shorr AF *et al*: **Telavancin versus vancomycin for hospital-acquired pneumonia due to gram-positive pathogens**. *Clin Infect Dis* 2011, **52**(1):31-40.

22. Aydemir H, Akduman D, Piskin N, Comert F, Horuz E, Terzi A, Kokturk F, Ornek T, Celebi G: **Colistin vs. the combination of colistin and rifampicin for the treatment of carbapenem-resistant Acinetobacter baumannii ventilator-associated pneumonia**. *Epidemiol Infect* 2013, **141**(6):1214-1222.

23. Kollef MH, Chastre J, Clavel M, Restrepo MI, Michiels B, Kaniga K, Cirillo I, Kimko H, Redman R: **A randomized trial of 7-day doripenem versus 10-day imipenem-cilastatin for ventilator-associated pneumonia**. *Crit Care* 2012, **16**(6):R218.

24. Wunderink RG, Niederman MS, Kollef MH, Shorr AF, Kunkel MJ, Baruch A, McGee WT, Reisman A, Chastre J: **Linezolid in methicillin-resistant Staphylococcus aureus nosocomial pneumonia: a randomized, controlled study**. *Clin Infect Dis* 2012, **54**(5):621-629.

25. Ramirez J, Dartois N, Gandjini H, Yan JL, Korth-Bradley J, McGovern PC: **Randomized phase 2 trial to evaluate the clinical efficacy of two high-dosage tigecycline regimens versus imipenem-cilastatin for treatment of hospital-acquired pneumonia**. *Antimicrob Agents Chemother* 2013, **57**(4):1756-1762.

26. Awad SS, Rodriguez AH, Chuang YC, Marjanek Z, Pareigis AJ, Reis G, Scheeren TW, Sanchez AS, Zhou X, Saulay M *et al*: **A phase 3 randomized double-blind comparison of ceftobiprole medocaril versus ceftazidime plus linezolid for the treatment of hospital-acquired pneumonia**. *Clin Infect Dis* 2014, **59**(1):51-61.

27. Kollef MH, Ricard JD, Roux D, Francois B, Ischaki E, Rozgonyi Z, Boulain T, Ivanyi Z, Janos G, Garot D *et al*: **A randomized trial of the amikacin fosfomycin inhalation system for the adjunctive therapy of Gram-negative ventilator-associated pneumonia: IASIS Trial**. *Chest* 2016.
